# Cardiovascular Risk Factor Analysis in Patients with a Recent Clinical Fracture at the Fracture Liaison Service

**DOI:** 10.1155/2014/710945

**Published:** 2014-08-27

**Authors:** Caroline E. Wyers, Lisanne Vranken, Robert Y. van der Velde, Piet P. M. M. Geusens, Heinrich M. J. Janzing, J. Wim Morrenhof, Joop P. W. van den Bergh

**Affiliations:** ^1^Department of Internal Medicine, VieCuri Medical Centre, P.O. Box 1926, 5900 BX Venlo, The Netherlands; ^2^Department of Internal Medicine, NUTRIM School for Nutrition, Toxicology and Metabolism, Maastricht University Medical Centre (MUMC), P.O. Box 616, 6200 MD Maastricht, The Netherlands; ^3^Department of Internal Medicine, Subdivision Rheumatology, CAPHRI, Maastricht University Medical Centre (MUMC), P.O. Box 616, 6200 MD Maastricht, The Netherlands; ^4^Biomedical Research Centre, Hasselt University, Agoralaan, Gebouw D, 3590 Diepenbeek, Belgium; ^5^Department of Surgery, VieCuri Medical Centre, P.O. Box 1926, 5900 BX Venlo, The Netherlands; ^6^Department of Orthopaedic Surgery, VieCuri Medical Centre, P.O. Box 1926, 5900 BX Venlo, The Netherlands

## Abstract

Patients with a low bone mineral density have an increased risk of cardiovascular diseases (CVD) and venous thromboembolic events (VTE). The aim of our retrospective chart review was to investigate the prevalence of CVD, VTE, hypertension (HT), and diabetes mellitus type 2 (DM2) in patients with a recent clinical fracture visiting the Fracture Liaison Service (FLS). Out of 3057 patients aged 50–90 years, 1359 consecutive patients, who agreed and were able to visit the FLS for fracture risk evaluation, were included (71.7% women; mean age 65.2 yrs). Based on medical history, 29.9% had a history of CVD (13.7%), VTE (1.7%), HT (14.9%), and DM2 (7.1%) or a combination. Their prevalence increased with age (21% in patients aged 50–59 years to 48% in patients aged >80 years) and was higher in men than in women (36% versus 27%), but independent of bone mineral density and fracture type. Careful evaluation of medical history with respect to these risk factors should be performed in patients with a recent clinical fracture before starting treatment with medications that increase the risk of VTE or cardiovascular events, such as raloxifene, strontium ranelate, or NSAIDs.

## 1. Introduction 

Osteoporosis and cardiovascular diseases (CVD) are two health care problems with a major impact on mortality and morbidity. In addition, the prevalence of both conditions increases as the population ages, and it is expected that the number of patients suffering from these conditions will rise in the future due to the increased life expectancy. Patients with a recent clinical fracture are screened and treated for osteoporosis, if necessary, at the Fracture Liaison Service (FLS) according to guidelines on osteoporosis and fracture prevention [1–6].

Patients with a low bone mineral density (BMD) have an increased risk for new cardiovascular events [7, 8] and low BMD is associated with more severe or advanced vascular calcification [9–16]. Postmenopausal women were reported to have an increased risk of cardiovascular events [17], with higher mortality [18], although in other studies these associations were not observed [19]. On the other hand, in patients diagnosed with a CVD, bone loss and fracture risk were increased [20–25].

The association between CVD and low BMD has clinical consequences for several therapies. Raloxifene is contraindicated in postmenopausal patients with a history of or an increased risk for venous thromboembolic events (VTE) [26, 27]. Nonsteroidal anti-inflammatory drugs (NSAIDs), prescribed for pain management, are contraindicated in patients with CVD or at risk of CVD including hypertension (HT), heart failure, and diabetes mellitus type 2 (DM2) [27, 28]. Strontium ranelate is contraindicated in patients with a history of cardiovascular diseases [29].

The aim of our retrospective chart review was therefore to investigate the prevalence of cardiovascular risk factors such as CVD, VTE, HT, and DM2 in medical history in patients at highest risk for a subsequent fracture, namely, those with a recent clinical fracture visiting the FLS.

## 2. Materials and Methods

### 2.1. Study Design and Population

This study was designed as a retrospective chart review to examine the prevalence of cardiovascular risk factors in postmenopausal women and men aged between 50 and 90 years with a recent clinical vertebral or nonvertebral fracture who were evaluated at FLS of VieCuri Medical Centre Noord-Limburg located in Venlo (The Netherlands). Patients with metastatic cancer in bone, fracture due to high energy trauma, or failure of prosthesis were excluded.

After primary fracture care, a specialised nurse in osteoporosis invited all patients with a recent clinical fracture to the FLS for screening for osteoporosis according to the Dutch guidelines [1]. Patients who agreed to be evaluated at the FLS received a detailed questionnaire for evaluation of risk factors for fractures, falls, detailed medical history including previous fractures and medication use, and daily dietary calcium intake. During the visit at the FLS, a trained nurse measured height and weight and evaluated the questionnaire with special attention to medical history and daily dietary calcium intake. In addition a BMD measurement with dual-energy X-ray absorptiometry (DXA) of the lumbar spine, total hip, and femoral neck was performed and a blood sample was collected to detect contributors to secondary osteoporosis and metabolic bone disease [30]. Depending on the results of BMD measurement, calcium intake, and serum 25-hydroxyvitamin D [25(OH)D] levels, patients were treated with adequate calcium intake, vitamin D supplements, and antiosteoporosis medication according to the Dutch guidelines for treatment of osteoporosis [1].

Fractures were classified according to Center et al. into hip fractures, major fractures (vertebra, multiple rib, humerus, pelvis, distal femur, and proximal tibia), minor fractures (all remaining fractures except fingers and toes), and finger and toe fractures [31].

### 2.2. Bone Densitometry

BMD in the hip and lumbar spine was measured using DXA with the Hologic QDR 4500 (Hologic, Bedford, MA, USA). Osteoporosis was diagnosed according to the WHO criteria for BMD [32]. Patients were classified according to the lowest value of* T*-score femoral neck, total hip, or lumbar spine.* T*-scores of ≤−2.5 standard deviations (SD) below the reference mean were classified as osteoporosis;* T*-scores between −1.0 and −2.5 SD were classified as osteopenia; and* T*-scores ≥−1.0 SD were classified as normal.

### 2.3. Cardiovascular Risk Factors

Medical history of all patients was systematically screened and cardiovascular risk factors were classified into CVD, VTE, HT, and DM2. CVD comprised ischaemic heart disease, myocardial infarction, angina pectoris, percutaneous coronary intervention, coronary bypass, cerebrovascular accident, transient ischaemic attack, and peripheral artery disease. VTE comprised venous thromboembolism and pulmonary embolism. In addition, patients were classified as having at least one cardiovascular risk factor if CVD or VTE or HT was present in medical history.

### 2.4. Statistical Analysis

Results are presented as means ± SD or percentages. Chi-square tests and Fisher's exact tests were used to test whether the variables are independent. Subgroup analyses were performed for gender, age per decade, BMD (normal versus osteopenia versus osteoporosis), and fracture type according to the center classification (finger and toe versus minor versus major versus hip). Logistic regression analyses were performed to adjust for age, sex, BMD (normal versus osteopenia versus osteoporosis), and fracture type according to the center classification (finger and toe versus minor versus major versus hip). All analyses were performed using SPSS for Mac (version 21.0, IBM SPSS Statistics, USA). A *P* value ≤ 0.05 was considered as statistically significant.

## 3. Results and Discussion

### 3.1. Study Population

From January 2009 until June 2011, 3131 patients aged between 50 and 90 years visited the emergency department with a recent clinical fracture. Seventy-four patients deceased before the invitation for fracture risk evaluation was sent, resulting in 3057 patients being invited. Of those, 1694 patients (55.4%) visited the FLS of whom 1359 (44.5%) had a fracture risk evaluation including BMD measurement. A total of 1359 patients (71.7% women and 28.3% men) with a mean age of 65.2 ± 9.5 years were evaluated at the FLS (Table 1). Osteoporosis was diagnosed in 29.6%, osteopenia was diagnosed in 47.7%, and 22.7% had a normal BMD. According to the center classification [31], 7.9% sustained a hip fracture, 28.7% a major fracture, 57.7% a minor fracture, and 5.7% a fracture of finger or toe. Based on medical history, 29.9% of the patients had a diagnosis of either CVD and/or VTE and/or hypertension and/or DM2. CVD was present in 13.7%, VTE in 1.7%, hypertension in 14.9%, and DM2 in 7.1% of patients visiting the FLS with a recent clinical fracture (Table 2).

### 3.2. Cardiovascular Risk Factors and Gender

The prevalence of CVD and/or VTE and/or hypertension and/or DM2 was significantly higher in men than in women (36.3% versus 27.3%; *P* = 0.001) (Table 2). CVD was more frequently diagnosed in men (*P* < 0.001), whereas the prevalence of VTE, HT, and DM2 was comparable for men and women. For the subcategories of CVD, myocardial infarction (*P* = 0.001), percutaneous coronary intervention (*P* < 0.001), and peripheral arterial disease (*P* = 0.001) were more frequently diagnosed in men. For other subcategories of CVD and for subcategories of VTE, HT, and DM2, the prevalence of those diseases was comparable between men and women.

### 3.3. Cardiovascular Risk Factors and Bone Mineral Density

There was no significant difference in the prevalence of CVD and/or VTE and/or HT and/or DM2 between patients with osteoporosis, osteopenia, and normal BMD (28.6%, 31.2%, and 29.1%, resp.; *P* = NS). Further, there was no significant difference in the prevalence of CVD, VTE, HT, DM2, and the subcategories of CVD and VTE (data not shown).

### 3.4. Cardiovascular Risk Factors and Fracture Type

**Figure 1:**
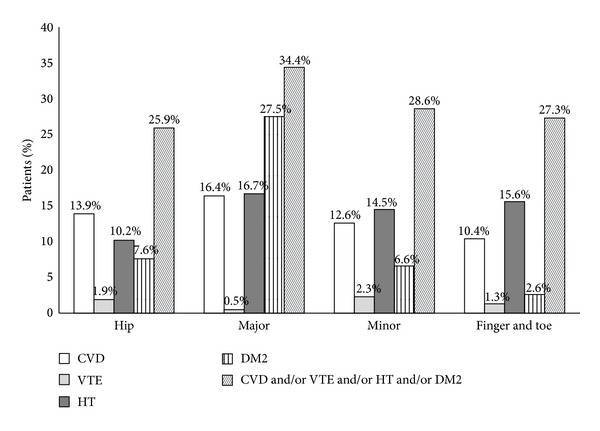
Prevalence of cardiovascular risk factors and diabetes mellitus type 2 according to the center classification.

As shown in Figure 1, in 34.4% of patients with a major fracture at least one cardiovascular risk factor or DM2 was present in medical history, as compared to 28.6% of patients with a minor fracture, 25.9% with a hip fracture, and 27.3% with a fracture of finger or toe (*P* = NS). In addition, there was no significant difference in the prevalence of CVD including its subcategories, VTE, HT, and DM2, if patients are classified according to fracture type. Only the prevalence of venous thromboembolism was significantly different (1.9% hip versus 0.5% major versus 2.3% minor versus 1.3% finger and toe; *P* = 0.029) (data not shown).

### 3.5. Cardiovascular Disease and Age

**Figure 2:**
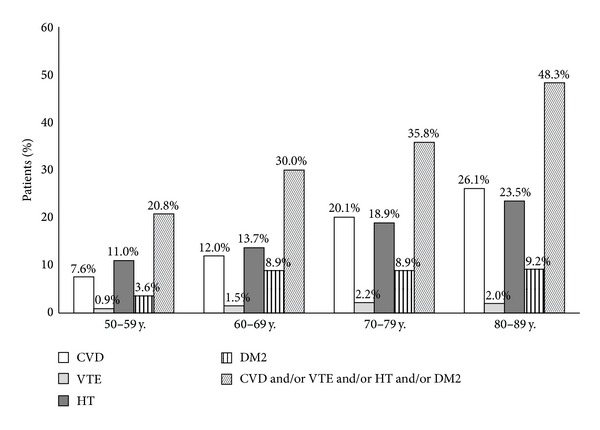
Prevalence of cardiovascular risk factors and diabetes mellitus type 2 according to age per decade.

As presented in Table 3 and in Figure 2, it is shown that the prevalence of CVD and/or VTE and/or HT and/or DM2 in medical history increased significantly with age, rising from 20.8% in patients aged 50–59 years to 48.3% in patients aged 80–89 years (*P* < 0.001). From the subgroups, CVD, HT, and DM2 increased significantly with age; CVD was present in 7.6% of patients aged 50–59 years up to 25.8% in patients aged 80–89 years (*P* = 0.006); HT 11.0% up to 23.3% (*P* = 0.001); and DM2 3.6% up to 23.3% (*P* = 0.006). For all subcategories of CVD except percutaneous coronary intervention, the prevalence increased significantly with age (Table 3). For VTE, only a significant increase was found for the presence of pulmonary embolism in medical history (*P* = 0.009).

In Table 4, it is shown that, for each decade except for the decade 80–89 years, the prevalence of cardiovascular risk factors is significantly higher in men as compared to women. Only in women and men aged between 60 and 69 years, the prevalence of having at least one cardiovascular risk factor and the prevalence of having at least one cardiovascular risk factor or DM2 is comparable between women and men (Table 4).

In addition, at least one of these conditions was present in medical history in 25.6% of patients aged 50–69 years and in 39.3% patients aged 70 years and older (*P* < 0.001). CVD, VTE, HT, or DM2 increased with age and was more frequently present in men as compared to women: 23.1% of women aged 50–69 years versus 35.6% of women aged 70 years and older (*P* < 0.001 within women) as compared to 31.2% of men aged 50–69 years versus 50.5% men aged 70 years and older (*P* < 0.001 within men) (data not shown).

### 3.6. Adjusted Analyses

After adjustments for age, sex, BMD, and fracture type, age and sex remained significant predictors for CVD (*P* < 0.001 for age; *P* < 0.001 for sex), age for VTE (*P* = 0.012), age and osteoporosis for HT (*P* < 0.001; *P* = 0.048 resp.), and age and osteoporosis for DM2 (*P* < 0.001 for age; *P* = 0.008 for osteoporosis).

In adjusted analyses only age and sex were significant predictors for the presence of at least one cardiovascular risk factor (CVD, VTE, or HT), (*P* < 0.001 for age; *P* < 0.001 for sex) and for the presence of at least one cardiovascular risk factor including DM2 (*P* < 0.001 for age; *P* < 0.001 for sex).

## 4. Discussion 

The aim of our retrospective review was to investigate the prevalence of cardiovascular risk factors including CVD, VTE, HT, and DM2 in medical history in patients with a recent clinical fracture visiting the FLS. Based on medical history, nearly one out of three patients had a medical history of CVD, VTE, HT, or DM2. CVD was more frequently present in men, whereas the prevalence of VTE, HT, and DM2 was similar in men and women. With increasing age, the prevalence of CVD, VTE, HT, and DM2 increased as well, up to half of men older than 70 years and of women older than 80 years.

There was no significant increase in the prevalence of these risk factors with decreasing BMD and increasing severity of fracture, except for BMD and HT and DM2. Adjusted analyses showed that age and sex remained significant predictors for the presence of CVD, VTE, HT, DM2, or at least one of these conditions, independent of BMD and fracture type according to the center classification and age and BMD for HT and DM2, independent of other risks.

The presence of cardiovascular risk factors in patients with a recent clinical fracture has important implications with regard to treatment and prevention of osteoporosis. Raloxifene is contraindicated in women with a history of VTE (including venous thromboembolism and pulmonary embolism) or women at risk of VTE [6, 33, 34], resulting in a contraindication in the prescription of raloxifene in 1.8% of women in our study. NSAIDs are contraindicated in patients with a history of CVD, heart failure, myocardial infarction, cerebrovascular accident, or transient ischaemic attack and in patients with an increased risk of ischaemic heart disease such as angina pectoris and percutaneous coronary disease and should be prescribed with caution in patients with HT and DM2 [28, 35], resulting in a contraindication for prescription of NSAIDs in 29.9% of patients (27.3% women versus 36.3% men). Recently, the EMA has advised to restrict the prescription of strontium ranelate in patients with a history of VTE, in patients at risk of VTE, and in patients with a CVD or HT in medical history [29], resulting in a contraindication for prescription of strontium ranelate in 26.5% of all patients (23.9% women versus 33.0% men).

Previous research has recommended that the treatment of cardiovascular disease should not only prevent new cardiovascular events, but also prevent fractures by evaluation and treatment of osteoporosis and vice versa [6].

This study has several limitations. First, the study is designed as a retrospective chart review. Therefore, we were not able to investigate the occurrence of new cardiovascular events after treatment with the antiosteoporosis medications was initiated. Second, only 55.4% of patients who visited the emergency department visited the FLS for fracture risk evaluation. Patients not visiting the FLS might be older, might have more severe fractures such as hip or humerus fractures for which surgical intervention was performed, might have postoperative complications, and might be living in a nursing home and are not able to visit the FLS. In combination with VTE often occurring after a major orthopaedic operation such as hip fracture surgery, the prevalence of cardiovascular risk factors might be underestimated.

## 5. Conclusions

In conclusion, CVD, VTE, HT, or DM2 was present in medical history of 29.9% of patients with a recent clinical fracture after age 50. The prevalence of these diseases increased with age and was higher in men than in women. These results emphasise that careful evaluation of medical history with respect to cardiovascular risk factors such as CVD, VTE, HT, and DM2 should be performed since medications such as raloxifene, strontium ranelate, and NSAIDs may increase cardiovascular risk or even may be contraindicated in a substantial number of patients with a recent clinical fracture.

## Figures and Tables

**Table 1:** Characteristics of the study population.

	Total (*n* = 1359)	Women (*n* = 974)	Men (*n* = 385)
Age, y. (SD)	65.2 (9.5)	65.6 (9.5)	64.2 (9.4)
Sex *n* (%)			
Women	974 (71.7)		
Men	385 (28.3)		
Weight, (kg)^a^	73.7 (14.6)	70.3 (13.5)	82.6 (13.4)
Height (m)^b^	1.68 (0.09)	1.64 (0.07)	1.76 (0.08)
BMI (kg/m^2^)^c^	26.3 (4.5)	26.1 (4.7)	26.7 (3.8)
Fracture location *n* (%)			
Hip	108 (7.9)	68 (7.0)	40 (10.4)
Major	390 (28.7)	281 (28.9)	109 (28.3)
Minor	784 (57.7)	571 (58.6)	213 (55.3)
Finger and toe	77 (5.7)	54 (5.5)	23 (6.0)
BMD *n* (%)			
Osteoporosis	402 (29.6)	329 (33.8)	73 (19.0)
Osteopenia	648 (47.7)	457 (46.9)	191 (49.6)
Normal BMD	309 (22.7)	188 (19.3)	121 (31.4)

^a^Weight was measured in 1194 patients (855 women, 339 men).

^b^Height was measured in 1237 patients (885 women, 352 men).

^c^BMI was calculated for 1150 (824 women, 326 men).

**Table 2:** Prevalence of cardiovascular risk factors and diabetes mellitus type 2 in patients presenting with a fracture after age 50.

	Total (*n* = 1359) *N* (%)	Women (*n* = 974) *N* (%)	Men (*n* = 385) *N* (%)	Value	df	*P* value
Cardiovascular disease (CVD)^a^	186 (13.7)	102 (10.5)	84 (21.8)	30.068	1	<0.001
Ischaemic heart disease	3 (0.2)	2 (0.2)	1 (0.3)			NS^b^
Myocardial infarction	39 (2.9)	19 (2.0)	20 (5.2)	10.418	1	0.001
Angina pectoris	27 (2.0)	18 (1.8)	9 (2.3)	0.340	1	NS
Percutaneous coronary intervention	33 (2.4)	12 (1.2)	21 (5.5)	20.765	1	<0.001
Coronary bypass	22 (1.6)	12 (1.2)	10 (2.6)	3.230	1	NS
Cerebrovascular accident	44 (3.2)	26 (2.7)	18 (4.7)	3.544	1	NS
Transient ischaemic attack	35 (2.6)	24 (2.5)	11 (2.9)	0.170	1	NS
Peripheral artery disease	40 (2.9)	19 (2.0)	21 (5.5)	11.858	1	0.001
Venous thromboembolic events (VTE)^c^	23 (1.7)	18 (1.8)	5 (1.3)	0.500	1	NS
Venous thromboembolism	15 (1.1)	13 (1.3)	2 (0.5)			NS
Pulmonary embolism	10 (0.7)	7 (0.7)	3 (0.8			NS
Hypertension (HT)	202 (14.9)	145 (14.9)	57 (14.8)	0.001	1	NS
Diabetes mellitus type 2 (DM2)	96 (7.1)	68 (7.0)	28 (7.3)	0.036	1	NS
CVD or VTE	202 (14.7)	112 (11.5)	88 (22.9)	28.362	1	<0.001
CVD or VTE or HT	360 (26.5)	233 (23.9)	127 (33.0)	11.644	1	0.001
CVD or VTE or HT or DM2	407 (29.9)	266 (27.3)	141 (36.3)	11.408	1	0.001

^a^Cardiovascular disease: having ischaemic heart disease or myocardial infarction or angina pectoris or percutaneous coronary intervention or bypass or cerebrovascular accident or transient ischaemic attack or peripheral artery disease in medical history.

^b^Fisher's exact test.

^c^Venous thromboembolic events: having venous thromboembolism or pulmonary thromboembolism in medical history.

**Table 3:** Prevalence of cardiovascular risk factors and diabetes mellitus type 2 according to age per decade in patients with a recent fracture after age 50.

	50–59 y. (*n* = 447) *N* (%)	60–69 y. (*n* = 474) *N* (%)	70–79 y. (*n* = 318) *N* (%)	80–89 y. (*n* = 120) *N* (%)	*P*-value^a^
Cardiovascular disease (CVD)^b^	34 (7.6)	57 (12.0)	64 (20.1)	31 (25.8)	0.006
Ischaemic heart disease	0 (0.0)	0 (0.0)	1 (0.3)	2 (1.7)	0.013
Myocardial infarction	5 (1.1)	17 (3.6)	12 (3.8)	5 (4.2)	0.030
Angina pectoris	1 (0.2)	5 (1.1)	14 (4.4)	7 (5.8)	<0.001
Percutaneous coronary intervention	6 (1.3)	14 (3.0)	9 (2.8)	4 (3.3)	NS
Coronary bypass	3 (0.7)	6 (1.3)	10 (3.1)	3 (2.5)	0.038
Cerebrovascular accident	10 (2.2)	6 (1.3)	17 (5.3)	11 (9.2)	<0.001
Transient ischaemic attack	7 (1.6)	10 (2.1)	10 (3.1)	8 (6.7)	0.024
Peripheral artery disease	7 (1.6)	13 (2.7)	13 (4.1)	7 (5.8)	0.040
Venous thromboembolic events (VTE)^c^	4 (0.9)	7 (1.5)	7 (2.2)	5 (4.2)	NS
Venous thromboembolism	3 (0.7)	6 (1.3)	4 (1.3)	2 (1.7)	NS
Pulmonary embolism	2 (0.4)	1 (0.2)	3 (0.9)	4 (3.3)	0.009
Hypertension (HT)	49 (11.0)	65 (13.7)	60 (18.9)	28 (23.3)	0.001
Diabetes mellitus type 2 (DM2)	16 (3.6)	42 (8.9)	27 (8.9)	11 (9.2)	0.006
CVD or VTE	37 (8.3)	62 (13.1)	67 (21.1)	34 (28.3)	<0.001
CVD or VTE or HT	81 (18.1)	120 (25.3)	105 (33.0)	54 (45.0)	<0.001
CVD or VTE or HT or DM2	93 (20.8)	142 (30.0)	114 (35.8)	58 (48.3)	<0.001

^a^Fisher's exact test.

^b^Cardiovascular disease: having ischaemic heart disease or myocardial infarction or angina pectoris or percutaneous coronary intervention or bypass or cerebrovascular accident or transient ischaemic attack or peripheral artery disease in medical history.

^c^Venous thromboembolic events: having venous thromboembolism or pulmonary thromboembolism in medical history.

**Table 4:** Prevalence of cardiovascular risk factors and diabetes mellitus type 2 according to age per decade and sex in patients with a recent fracture after age 50.

	50–59 y.			60–69 y.			70–79 y.			80–89 y.		
	Women (*n* = 302) *N* (%)	Men (*n* = 145) *N* (%)	*P* value	Women (*n* = 343) *N* (%)	Men (*n* = 131) *N* (%)	*P* value	Women (*n* = 237) *N* (%)	Men (*n* = 81) *N* (%)	*P* value	Women (*n* = 92) *N* (%)	Men (*n* = 28) *N* (%)	*P* value
Cardiovascular disease (CVD)^a^	12 (4.0)	22 (15.2)	<0.001	22 (4.0)	25 (19.1)	0.004	38 (16.0)	26 (32.1)	0.002	20 (21.7)	11 (39.3)	NS
Venous thromboembolic events (VTE)^b^	3 (1.0)	1 (0.7)	NS^c^	6 (1.7)	1 (0.8)	NS^c^	4 (1.7)	3 (3.7)	NS^c^	5 (5.4)	0 (0.0)	NS^c^
Hypertension (HT)	31 (10.3)	18 (12.4)	NS	49 (14.3)	16 (21.2)	NS	40 (16.9)	20 (24.7)	NS	25 (27.2)	3 (10.7)	NS
Diabetes mellitus type 2 (DM2)	11 (3.6)	5 (3.4)	NS	30 (8.7)	12 (9.2)	NS	18 (7.6)	9 (11.1)	NS	9 (9.8)	2 (7.1)	NS^c^
CVD or VTE	14 (4.6)	23 (15.9)	<0.001	36 (10.5)	26 (19.8)	0.007	39 (16.5)	28 (34.6)	0.001	23 (25.0)	11 (39.3)	NS
CVD or VTE or HT	42 (13.9)	39 (26.9)	0.001	82 (23.9)	38 (29.0)	NS	67 (28.3)	38 (46.9)	0.002	42 (46.7)	12 (42.9)	NS
CVD or VTE or HT or DM2	50 (16.6)	43 (29.7)	0.001	99 (28.9)	43 (32.8)	NS	72 (30.4)	42 (51.9)	0.001	45 (48.9)	13 (46.4)	NS

^a^Cardiovascular disease: having ischaemic heart disease or myocardial infarction or angina pectoris or percutaneous coronary intervention or bypass or cerebrovascular accident or transient ischaemic attack or peripheral artery disease in medical history.

^b^Venous thromboembolic events: having venous thromboembolism or pulmonary thromboembolism in medical history.

^c^Fisher's exact test.

## References

[1] Dutch Institute for Healthcare Improvement CBO (2011). *Richtlijn Osteoporose en Fractuurpreventie, Derde Herziening [Dutch]*.

[2] Eisman JA, Bogoch ER, Dell R (2012). Making the first fracture the last fracture: ASBMR task force report on secondary fracture prevention. *Journal of Bone and Mineral Research*.

[3] McLellan AR, Wolowacz SE, Zimovetz EA (2011). Fracture liaison services for the evaluation and management of patients with osteoporotic fracture: a cost-effectiveness evaluation based on data collected over 8 years of service provision. *Osteoporosis International*.

[4] Compston J, Bowring C, Cooper A (2013). Diagnosis and management of osteoporosis in postmenopausal women and older men in the UK: National Osteoporosis Guideline Group (NOGG) update 2013. *Maturitas*.

[5] Compston J, Cooper A, Cooper C (2009). Guidelines for the diagnosis and management of osteoporosis in postmenopausal women and men from the age of 50 years in the UK. *Maturitas*.

[6] Kanis JA, McCloskey EV, Johansson H, Cooper C, Rizzoli R, Reginster J-Y (2013). European guidance for the diagnosis and management of osteoporosis in postmenopausal women. *Osteoporosis International*.

[7] Farhat GN, Newman AB, Sutton-Tyrrell K (2007). The association of bone mineral density measures with incident cardiovascular disease in older adults. *Osteoporosis International*.

[8] Farhat GN, Cauley JA (2008). The link between osteoporosis and cardiovascular disease. *Clinical Cases in Mineral and Bone Metabolism*.

[9] Hyder JA, Allison MA, Criqui MH, Wright CM (2007). Association between systemic calcified atherosclerosis and bone density. *Calcified Tissue International*.

[10] Choi SH, An JH, Lim S (2009). Lower bone mineral density is associated with higher coronary calcification and coronary plaque burdens by multidetector row coronary computed tomography in pre- and postmenopausal women. *Clinical Endocrinology*.

[11] Hak AE, Pols HAP, van Hemert AM, Hofman A, Witteman JCM (2000). Progression of aortic calcification is associated with metacarpal bone loss during menopause: a population-based longitudinal study. *Arteriosclerosis, Thrombosis, and Vascular Biology*.

[12] Tankó LB, Bagger YZ, Christiansen C (2003). Low bone mineral density in the hip as a marker of advanced atherosclerosis in elderly women. *Calcified Tissue International*.

[13] Uyama O, Yoshimoto Y, Yamamoto Y, Kawai A (1997). Bone changes and carotid atherosclerosis in postmenopausal women. *Stroke*.

[14] Seo SK, Cho S, Kim HY (2009). Bone mineral density, arterial stiffness, and coronary atherosclerosis in healthy postmenopausal women. *Menopause*.

[15] Sumino H, Ichikawa S, Kasama S (2008). Relationship between carotid atherosclerosis and lumbar spine bone mineral density in postmenopausal women. *Hypertension Research*.

[16] Lampropoulos CE, Papaioannou I, D'Cruz DP (2012). Osteoporosis—a risk factor for cardiovascular disease?. *Nature Reviews Rheumatology*.

[17] Tanko LB, Christiansen C, Cox DA, Geiger MJ, McNabb MA, Cummings SR (2005). Relationship between osteoporosis and cardiovascular disease in postmenopausal women. *Journal of Bone and Mineral Research*.

[18] Mussolino ME, Madans JH, Gillum RF (2003). Bone mineral density and mortality in women and men: the NHANES I epidemiologic follow-up study. *Annals of Epidemiology*.

[19] Mussolino ME, Armenian HK (2007). Low Bone Mineral Density, Coronary Heart Disease, and Stroke Mortality in Men and Women: The Third National Health and Nutrition Examination Survey. *Annals of Epidemiology*.

[20] Chen JS, Hogan C, Lyubomirsky G, Sambrook PN (2011). Women with cardiovascular disease have increased risk of osteoporotic fracture. *Calcified Tissue International*.

[21] Lyons KJ, Majumdar SR, Ezekowitz JA (2011). The unrecognized burden of osteoporosis-related vertebral fractures in patients with heart failure. *Circulation: Heart Failure*.

[22] Sennerby U, Farahmand B, Ahlbom A, Ljunghall S, Michaëlsson K (2007). Cardiovascular diseases and future risk of hip fracture in women. *Osteoporosis International*.

[23] van Diepen S, Majumdar SR, Bakal JA, McAlister FA, Ezekowitz JA (2008). Heart failure is a risk factor for orthopedic fracture: a population-based analysis of 16 294 patients. *Circulation*.

[24] Vestergaard P, Rejnmark L, Mosekilde L (2009). Hypertension is a risk factor for fractures. *Calcified Tissue International*.

[25] den Uyl D, Nurmohamed MT, van Tuyl LHD, Raterman HG, Lems WF (2011). (Sub)clinical cardiovascular disease is associated with increased bone loss and fracture risk; A systematic review of the association between cardiovascular disease and osteoporosis. *Arthritis Research and Therapy*.

[26] Lippuner K, Buchard PA, de Geyter C (2012). Recommendations for raloxifene use in daily clinical practice in the Swiss setting. *European Spine Journal*.

[27] Goldstein SR, Duvernoy CS, Calaf J (2009). Raloxifene use in clinical practice: efficacy and safety. *Menopause*.

[28] Ray WA, Stein CM, Hall K, Daugherty JR, Griffin MR (2002). Non-steroidal anti-inflammatory drugs and risk of serious coronary heart disease: an observational cohort study. *The Lancet*.

[29] European Medicines Agency (2013). *PSUR Assessment Report Strontium Ranelate*.

[30] Bours SPG, Van Geel TACM, Geusens PPMM (2011). Contributors to secondary osteoporosis and metabolic bone diseases in patients presenting with a clinical fracture. *The Journal of Clinical Endocrinology and Metabolism*.

[31] Center JR, Bliuc D, Nguyen TV, Eisman JA (2007). Risk of subsequent fracture after low-trauma fracture in men and women. *Journal of the American Medical Association*.

[32] World Health Organisation (2003). Prevention and management of osteoporosis. *World Health Organization Technical Report Series*.

[33] Barrett-Connor E, Mosca L, Collins P (2006). Effects of raloxifene on cardiovascular events and breast cancer in postmenopausal women. *New England Journal of Medicine*.

[34] Duvernoy CS, Yeo AA, Wong M, Cox DA, Kim HM (2010). Antiplatelet therapy use and the risk of venous thromboembolic events in the raloxifene use for the heart (RUTH) trial. *Journal of Women's Health*.

[35] Vonkeman HE, Brouwers JRBJ, van de Laar MAFJ (2006). Understanding the NSAID related risk of vascular events. *British Medical Journal*.

